# Lung Ultrasound Findings Compared with Chest X-Ray Findings in Known Pulmonary Tuberculosis Patients: A Cross-Sectional Study in Lima, Peru

**DOI:** 10.4269/ajtmh.20-0542

**Published:** 2020-08-17

**Authors:** Matthew Fentress, Cesar Ugarte-Gil, Miguel Cervantes, Diego Rivas, David Moore, Philip Caliguiri, Kevin Bergman, Sassan Noazin, Andrew Padovani, Robert H. Gilman

**Affiliations:** 1 University of California, Davis, Sacramento, California;; 2 Bloomberg School of Public Health, Johns Hopkins University, Baltimore, Maryland;; 3 Universidad Peruana Cayetano Heredia, Lima, Peru;; 4 London School of Hygiene and Tropical Medicine, London, United Kingdom;; 5 Associacion Benefica PRISMA, Lima, Peru;; 6 University of Utah, Salt Lake City, Utah;; 7 Contra Costa Family Medicine Residency, University of California - San Francisco, Martinez, California

## Abstract

Lung ultrasound (LUS) is highly portable and has excellent diagnostic accuracy for pneumonia compared with conventional radiography, but the literature on its use in pulmonary tuberculosis (PTB) is limited. This study characterized LUS lesions in patients with PTB and compared them with chest X-ray (CXR) findings. Adult patients in Lima, Peru, with PTB were recruited within 1 week of starting antituberculosis treatment. Comprehensive LUS was performed in all patients at enrollment and assessed for consolidation, small subpleural consolidation (SPC, hypothesized to be a marker of CXR consolidation), cavity, pleural effusion, pathologic B-lines, and miliary pattern. Patient CXRs were digitized and interpreted by a board-certified radiologist. Fifty-one patients were included in the final analysis. Lung ultrasound detected either consolidation or SPC in 96.1% of participants. No significant difference was found between the LUS detection of a composite of consolidation or SPC, and CXR detection of consolidation (96.1% versus 98%, *P* > 0.99). The proportion of patients with cavity detected by LUS was significantly lower than that detected by CXR (5.9% versus 51%, *P* < 0.001). Overall, LUS detection of consolidation or SPC may be a sensitive marker for diagnosis of PTB. Lung ultrasound demonstrated poor ability to detect radiographically identified cavity, although previous studies suggest SPC could add specificity for the diagnosis of PTB. Based on its portability and evidence base for diagnosing other pulmonary diseases, LUS may have a role in screening and diagnosis of PTB in areas without ready access to CXR. Further studies should evaluate its diagnostic accuracy in patients with and without PTB.

## INTRODUCTION

More than 10 million people were diagnosed with active tuberculosis in 2018, and an estimated 1.5 million died of the disease in the same year.^1^ Timely and accurate diagnosis can lead to improved patient outcomes and enhance tuberculosis control efforts, as undiagnosed patients may continue to infect others in the community until they are appropriately diagnosed and treated. Current diagnostic algorithms for pulmonary tuberculosis (PTB) rely primarily on sputum microscopy, chest X-ray (CXR), and, more recently, nucleic acid amplification tests (NAAT). However, microscopy has relatively poor sensitivity,^2^ and many clinicians in resource-limited areas where PTB is common may not have ready access to CXR or NAAT. Improved point-of-care diagnostics for tuberculosis have been identified as a research priority by the WHO.^3^ Lung ultrasound (LUS) is highly portable, can be performed at the bedside, and has excellent performance characteristics for diagnosis of pneumonia compared with conventional chest imaging (sensitivity 95%, specificity 97%).^4–7^ If diagnostic accuracy of LUS for PTB is similar to that seen for pneumonia, it could play an important role in screening and diagnosis of PTB in areas without ready access to CXR. Despite the extensive literature base for LUS in pneumonia, the literature on its use for diagnosis of PTB is limited,^8–12^ and available studies have methodological limitations including small size and lack of systematic comparison to a diagnostic imaging gold standard. This study therefore sought to systematically characterize LUS findings in adult patients with confirmed PTB and compare these LUS findings with CXR findings.

## MATERIALS AND METHODS

A cross-sectional study was conducted among ambulatory patients in Lima, Peru. The inclusion criteria included age ≥ 18 years; diagnosis of PTB by positive sputum smear for acid fast bacilli (AFB), positive sputum PCR, or positive culture for *Mycobacterium tuberculosis*; and initiation of treatment for PTB within 1 week. The exclusion criteria included inability to cooperate with the LUS protocol or inability to provide informed consent. The incidence of tuberculosis in Peru is 116/100,000, representing one of the highest incidence rates in the Americas, and the incidence of HIV/tuberculosis (TB) coinfection is 5.7/100,000.^13^ Ethical approval was obtained from the London School of Hygiene and Tropical Medicine, Associacion Benefica PRISMA (Peru), and Hospital Huaycan (Peru). Government approval was obtained from the Peru Ministry of Health.

**Figure 1.**
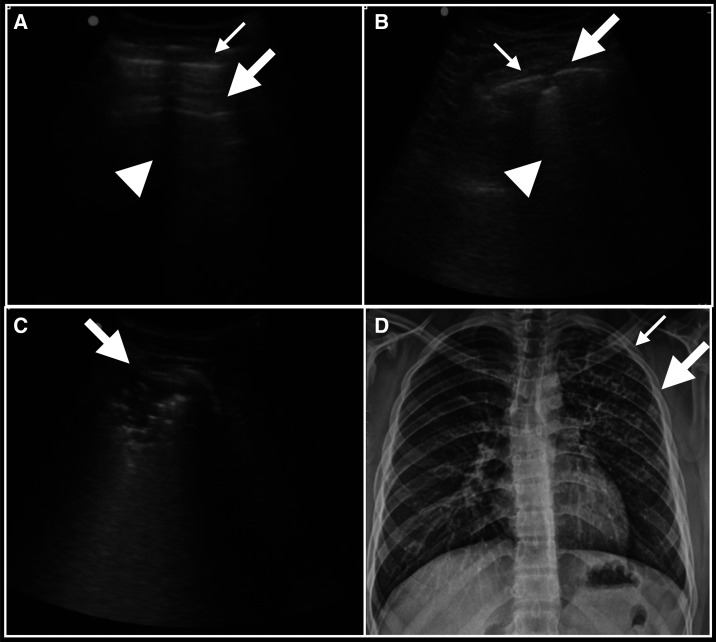
Typical appearance of lesions in lung ultrasound (LUS) and chest X-ray from a pulmonary tuberculosis (PTB) patient: (**A**) *Normal* LUS with white pleural line (thin arrow) and A-line reverberation artifact (thick arrow), indicative of normal air-filled pulmonary parenchyma. Rib shadow (arrow head) in the center. (**B**) Typical appearance of small subpleural consolidation (SPC) (thick arrow),characterized by subpleural hypoechoic region < 1 × 1 cm, with distinct borders and trailing artifact (arrowhead), next to the normal white pleural line (thin arrow). (**C**) Typical appearance of consolidation (thick arrow), characterized by echo-poor region > 1 × 1 cm, with or without air bronchograms. Air bronchograms seen here as hyperechoic spots within the consolidation. (**D**) Chest X-ray from a PTB patient demonstrates patchy fibronodular left upper lung field consolidation (thick arrow) and cavity (thin arrow). Lung ultrasound of the same patient showed multiple SPCs throughout the left upper and middle hemithorax, and a 3.5 × 1.2-cm consolidation in the left middle lung field.

Fifty-three patients were recruited between January 2019 and December 2019. Two patients were excluded because of inconsistent or incomplete data. Demographic and clinical data were collected from the medical record and enrollment questionnaire. Lung ultrasound was performed with a Sonosite Micromaxx (FUJIFILM Sonosite, Bothell, WA) using a 5-2 MHz curvilinear probe. Each intercostal space was systematically interrogated from apices to diaphragm in transverse and longitudinal planes according to previously described techniques.^9,14^ Each hemithorax was imaged in anterior, lateral, and posterior zones and findings recorded for three regions within each zone—upper, middle, and lower—for data capture in a total of 18 regions per participant. Each region was classified based on the presence or absence of six abnormal LUS findings: consolidation, small subpleural consolidation (SPC), cavity, pleural effusion, pathologic B-lines, or miliary pattern (Table 1, Figure 1). Examinations were performed primarily by two Peruvian general practitioners (MC and DR) following a 30-hour training; two examinations were performed by a physician with experience in more than 100 LUS examinations (MF). Clinicians performing the LUS examinations were blinded to the patients’ CXR findings. Representative LUS clips were saved from each region, and 50% of randomly selected patients’ images were reviewed by an LUS expert.

**Table 1:** Classification of lung ultrasound findings

Ultrasound finding	Definition
Small subpleural consolidation	Subpleural, nodular, hypoechoic region < 1 × 1 cm, with distinct borders and trailing comet-tail artifacts
Consolidation	Subpleural, echo-poor or tissue-like region > 1 cm, with or without sonographic air bronchograms
Miliary pattern	Diffuse, bilateral pattern of multiple B-lines and subpleural sonographic granularity
Cavitation	Consolidation > 1 cm with hypoechoic central clearing
Pleural effusion	Free pleural fluid
Abnormal B-line pattern	Vertical, hyperechoic reverberation artifacts which arise from the pleural line, extend to the bottom of the screen, and move with respiration (30% or greater of probe footprint considered abnormal)

Chest X-rays performed as part of routine medical care were reviewed by a radiologist and classified based on the presence and location of consolidations, cavities, pleural effusions, and miliary pattern. Percentage of CXR affected was assessed by dividing the number of pixels in hand-drawn regions of interest around abnormalities anywhere on the CXRs (using the freeware GNU Image Manipulation Program v. 2.10.10) by the number of total pixels of hand-drawn regions of both the whole right and the whole left lungs, according to a previously published method.^15^ This software analysis was only performed on CXRs and not on LUS.

Descriptive statistics were used to characterize demographic and clinical data, type and location of sonographic abnormalities, and type and location of CXR abnormalities. Similar analyses were used to characterize an LUS composite of consolidation or SPC, hypothesized to be a potential marker of CXR consolidation. Detection of lesions by the two imaging modalities was compared using the exact McNemar test. Linear regression (ordinary least squares) performed after log transformation was used to analyze the association between percentage of CXR affected and percentage of LUS regions in which either consolidation or SPC was detected. Agreement between field and expert interpretation of LUS was analyzed by Cohen’s kappa coefficient, and LUS image quality was assessed on a 5-point Likert quality scale graded by the external ultrasound expert.

## RESULTS

The final analysis included 51 patients with PTB. The mean age of participants was 34 (SD 15.8, range 18–78) years. Females accounted for 31.4% of the participants, 27.5% were smokers, mean body mass index was 22.6. Overall, 98% of participants were AFB smear positive, and none were HIV positive (Table 2).

**Table 2:** Patient demographics and clinical data

	***N***	**Mean**	SD	Minimum	Maximum
Age (years)	51	33.7	15.81	18	78
Gender	*N*	**Share (%)**			
Male	35	68.6	–	–	–
Female	16	31.4	–	–	–
Risk factors					
HIV positive	0	0.0	–	–	–
Tuberculosis contact	13	25.5	–	–	–
Smoker	14	27.5	–	–	–
Bacille Calmette-Guerin (BCG) status	50	98.0	–	–	–
Weight (kg)	***N***	**Mean**	SD	Minimum	Maximum
Baseline	46	58.3	9.9	41.5	87
BMI	45	22.6	3.9	16.6	33.7
Symptoms, previous 2 weeks	*N*	**Share (%)**			
Fever	38	74.5	–	–	–
Cough	49	96.1	–	–	–
Hemoptysis	32	62.7	–	–	–
Night sweats	27	52.9	–	–	–
Weight loss	43	84.3	–	–	–
Fatigue	41	80.4	–	–	–

No significant difference was found between the proportion of participants with the LUS composite of consolidation or SPC, hypothesized to be a marker of radiographic consolidation, and CXR consolidation (96.1% versus 98%, *P* > 0.99). Sonographic consolidation was detected in 80.4% (95% CI: 66.7–89.3) of participants, SPC in 80.4%, cavity in 5.9% (95% CI: 1.8–17.3), pleural effusion in 7.8% (95% CI: 2.9–19.7), pathologic B-line in 39.2% (95% CI: 26.5–53.6), and miliary pattern in none. Sonographic consolidation was detected in the upper fields in 62.7% of participants, in the middle fields in 56.9%, in the lower fields in 21.6%, and bilaterally in 35.3%. SPC was detected in the upper fields in 52.9% of participants, in the middle fields in 52.9%, in the lower fields in 27.5%, and bilaterally in 31.4%. The composite LUS finding of either consolidation or SPC was detected in 96.1% (95% CI: 85.0–99.1) of participants, including 80.4% in the upper fields, 74.5% in the middle fields, and 43.1% in the lower fields (Tables 3 and 4).

**Table 3:** Type and location of lung ultrasound lesions in pulmonary tuberculosis patients

	All fields			Location									Laterality								
				Upper			Middle			Lower			Right			Left			Bilateral		
	No.	Share (%)	95% CI for share	No.	Share (%)	95% CI for share	No.	Share (%)	95% CI for share	No.	Share (%)	95% CI for share	No.	Share (%)	95% CI for share	No.	Share (%)	95% CI for share	No.	Share (%)	95% CI for share
Consolidation	41	80.4	(66.72–89.34)	32	62.7	(48.35–75.19)	29	56.9	(42.62–70.05)	11	21.6	(12.11–35.43)	30	58.8	(44.51–71.79)	29	56.9	(42.62–70.05)	18	35.3	(23.14–49.71)
SPC	41	80.4	(66.72–89.34)	27	52.9	(38.90–66.53)	27	52.9	(38.90–66.53)	14	27.5	(16.68–41.69)	30	58.8	(44.51–71.79)	27	52.9	(38.90–66.53)	16	31.4	(19.86–45.75)
Cavity	3	5.9	(1.83–17.29)	–	–	–	3	5.9	(1.83–17.29)	–	–	–	2	3.9	(0.94–14.99)	1	2	(0.26–13.43)	–	–	–
Pleural effusion	4	7.8	–	–	–	–	–	–	–	4	7.8	(2.87–19.67)	4	7.8	–	–	–	–	–	–	–
Pathological B-line	20	39.2	(26.50–53.58)	4	7.9	(2.87–19.67)	11	21.6	(12.11–35.43)	13	25.5	(15.13–39.63)	18	35.3	(23.14–49.71)	7	13.7	(6.51–26.64)	5	9.8	(4.01–22.03)
Miliary pattern	–	–	–	–	–	–	–	–	–	–	–	–	–	–	–	–	–	–	–	–	–
Consolidation or SPC	49	96.1	(85.01–99.06)	41	80.4	(66.72–89.34)	38	74.5	(60.37–84.87)	22	43.1	(29.95–57.38)	41	80.4	(66.72–89.34)	38	74.5	(60.37–84.87)	25	49	(35.26–62.92)

SPC = small subpleural consolidation.

**Table 4:** Type, location, and number of lung ultrasound lesions in pulmonary tuberculosis patients

	Anterior			Lateral			Posterior			Lesions per patient	
	No.	Share (%)	95% CI for share	No.	Share (%)	95% CI for share	No.	Share (%)	95% CI for share	Mean	S.E.
Consolidation	26	51	(37.1–64.7)	18	35.3	(23.1–49.7)	34	66.7	(52.3–78.5)	2.68	1.57
SPC	32	62.7	(48.3–75.2)	20	39.2	(26.5–53.6)	22	43.1	(29.9–57.4)	2.76	1.81
Cavity	1	2	(0.3–13.4)	–	–	(0.9–15.0)	2	3.9	–	1	0
Pleural effusion	1	2	(0.3–13.4)	2	3.9	(0.9–15.0)	1	2	(0.3–13.4)	1	0
Pathological B-line	8	15.7	(7.9–28.9)	10	19.6	(10.7–33.3)	9	17.6	(9.2–31.1)	1.55	0.69
Miliary pattern	–	–	–	–	–	–	–	–	–	–	–
Consolidation or SPC	41	80.4	(66.7–89.3)	31	60.8	(46.4–73.5)	44	86.3	(73.4–93.5)	4.55	2.91

SPC = small subpleural consolidation.

Radiographic consolidation was detected in 98% (95% CI: 86.6–99.7) of participants, cavity in 51% (95% CI: 37.1–64.7), pleural effusion in 3.9% (95% CI: 0.9–15.0), and miliary pattern in none. Chest X-ray detected consolidation in the upper fields in 74.5% of participants, middle fields in 45.1%, lower fields in 21.6%, and bilaterally in 23.5%. Chest X-ray detected 58 individual cavities in 26 patients. Chest X-ray detected cavity in the upper fields in 43.1% of participants, middle fields in 9.8%, lower fields in 2%, and bilaterally in 2% (Table 5).

**Table 5:** Type and location of chest X-ray lesions seen in pulmonary tuberculosis patients

				Location									Laterality								
	All fields			Upper			Middle			Lower			Right			Left			Bilateral		
	No.	Share (%)	95% CI for share	No.	Share (%)	95% CI for share	No.	Share (%)	95% CI for share	No.	Share (%)	95% CI for share	No.	Share (%)	95% CI for share	No.	Share (%)	95% CI for share	No.	Share (%)	95% CI for share
Consolidation	50	98	(86.6–99.7)	38	74.5	(60.4–84.9)	23	45.1	(31.7–59.2)	11	21.6	(12.1–35.4)	33	64.7	(50.3–76.9)	29	56.9	(42.6–70.1)	12	23.5	(13.6–37.5)
Cavity	26	51	(37.1–64.7)	22	43.1	(29.9–57.4)	5	9.8	(4.0–22.0)	1	2	(0.3–13.4)	17	33.3	(21.5–47.7)	10	19.6	(10.7–33.3)	1	2	(0.3–13.4)
Pleural effusion	2	3.9	(0.9–15.0)	–	–	–	–	–	–	–	–	–	2	3.9	(0.9–15.0)	–	–	–	–	–	–
Miliary pattern	–	–	–	–	–	–	–	–	–	–	–	–	–	–	–	–	–	–	–	–	–
Consolidation or cavity	51	100	–	40	78.4	(64.6–87.9)	24	47.1	(33.5–61.1)	11	21.6	(12.1–35.4)	34	66.7	(52.3–78.5)	31	60.8	(46.4–73.5)	12	23.5	(13.6–37.5)

**Figure 2.**
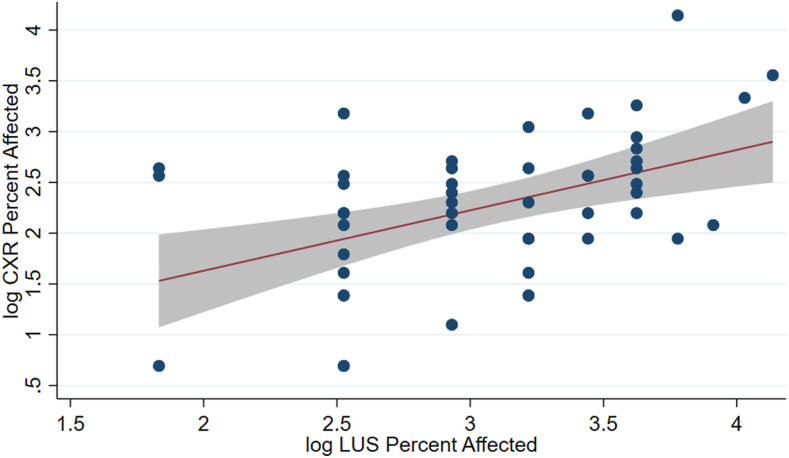
Relationship between chest X-ray percent affected and lung ultrasound percent affected. Coefficient 0.593 (0.197–0.993), intercept 0.44. The gray area represents a 95% CI around the fitted line. Lung ultrasound percent affected is calculated as the ratio of the number of fields with consolidations or small subpleural consolidation to the total number of fields. This figure appears in color at www.ajtmh.org.

Chest X-ray detected consolidation in a significantly higher proportion of patients than LUS (98% versus 80.4%, *P* = 0.004). Lung ultrasound composite of consolidation or SPC was detected in a significantly higher proportion of patients than CXR consolidation in the middle (56.9% versus 45.1%, *P* = 0.004) and lower (43.1% versus 21.6%, *P* = 0.035) lung fields, and bilaterally (49% versus 23.5%, *P* = 0.015), although other anatomic locations showed no significant difference. Chest X-ray detected cavity in a significantly higher proportion of patients than LUS (51% versus 5.9%, *P* < 0.001). Linear regression showed that a 1% change in percentage of regions affected on LUS was associated with a 0.6% change in percentage of CXR affected (*P* < 0.01, *R*^2^ = 0.215, Figure 2).

Correlation between study sonographers’ interpretation and expert reviewer interpretation was excellent (kappa coefficient = 0.93). The average LUS image quality, as assessed by external expert review on a 5-point Likert scale with 1 being poor and 5 being excellent, was 4.8 (SD 0.39).

## DISCUSSION

This is the first study to our knowledge to systematically compare parenchymal LUS and CXR findings in adult PTB patients. A high proportion (96.1%) of patients with PTB in our cohort had the composite of either consolidation or SPC detected on LUS. These findings are similar to those reported in previous studies. Agostinis^10^ found 97% of PTB patients had small subpleural lesions (termed “subpleural nodules” in that study, which appear morphologically identical to SPCs), whereas Montouri^9^ found 73% of PTB patients had small subpleural lesions and 77% had consolidations. The ability of LUS in the present study to detect radiographically identified cavity was very poor, and this is a major limitation of the tool. However, only two patients in our study had no consolidation or SPC on LUS, and each of these participants had subtle, proximally located, non-cavitary lesions on CXR. Therefore, no patients with cavitary disease would have been missed if the LUS composite of consolidation or SPC were used as a screening tool in the study group.

These findings indicate that the presence of an LUS composite of consolidation or SPC has high sensitivity for PTB. Although we cannot describe specificity with the current model, previous studies suggest the presence of SPC may add specificity for the diagnosis of PTB, with a reported specificity of 67%.^9^ The specificity of CXR for diagnosis of PTB reported in the literature is similar, ranging from 63% to 67%.^16,17^

Although SPCs—subpleural, nodular hypoechoic lesions typically < 1 × 1 cm —are described in other conditions such as pulmonary embolism, viral pneumonia including COVID-19, pneumocystis pneumonia, and early bacterial pneumonia, our data show that these sonographic lesions are frequently encountered in the patchy fibronodular infiltrates commonly seen in PTB. Lung ultrasound detects lesions that touch the pleural line,^18^ and most acute pulmonary disorders reach the pleural line,^19^ including 98.5% of acute consolidations seen in critically ill patients by chest computed tomography.^20^ Typically, where these consolidations touch a large segment of the pleura, they produce the sonographic appearance of a consolidation. However, patchy or fibronodular consolidations such as those often found in PTB may contact the pleural surface in only a patchy or limited manner. A review of the LUS and CXR images from this study suggests that where radiographic lesions touch the pleura in a patchy or limited manner, they may produce the sonographic appearance of an SPC rather than a consolidation (Figure 1). This suggests that SPCs could be a potential marker for CXR consolidation in PTB, particularly if that CXR consolidation is patchy, irregular, or small in size.

Furthermore, LUS composite of either consolidation or SPC was detected in a significantly higher proportion of patients than CXR consolidation in several anatomic locations (middle field, lower field, and bilaterally). This suggests LUS may be able to detect subtle lesions not appreciated on CXR in PTB patients.

The selection of LUS findings assessed in this study was based largely on findings in PTB patients from previous studies.^9,10,21^ Small subpleural consolidations were initially described in PTB patients by Agostinis^10^ in 2017, then by Montuori^9^ in 2019, although both referred to these lesions as “subpleural nodules” and abbreviated the term to “SUN.” Sonographic consolidations in PTB patients were first described by Heuvlings^22^ in 2016, and subsequently by other authors.^9,10^ The miliary pattern was first described by Hunter^21^ in a case series of 10 miliary PTB patients in South Africa. Cavitation in PTB patients has been previously described by Agostinis^10^ and Montuori.^9^ Each reported them in only a small proportion of patients, and Montuori described poor cavity detection by LUS even when using a non-blinded technique. Pathologic B-lines, to our knowledge, have not previously been reported in the PTB literature, although they are widely described in the LUS literature as a sign of interstitial edema.^23^ We did not observe a clear trend in the data that would indicate pathologic B-lines were associated with consolidations in PTB, although this was not analyzed at an individual case level and could be explored further in subsequent studies. Pleural effusions have been described in PTB patients in numerous studies.^8^ Some LUS studies have also included an assessment for pleural irregularities, although we chose to omit this classification as the finding is often subtle and may be difficult to distinguish from a small SPC.

The poor cavity detection by LUS in this study is similar to the experiences described by Agostinis^10^ and Montuori.^9^ In our study, a total of 58 individual cavities in 26 patients were identified on CXR—and, therefore, potentially could have been detected by LUS—although LUS only detected three individual cavities. The poor cavity detection in this study could be due to limitations of the technique, which used a low-frequency curvilinear probe without color flow and did not use a high-frequency (i.e., high resolution) linear probe; or due to limitations of the machine, which is an older model no longer in production. If this is the case, detection may improve as ultrasound equipment and resolution improve. However, it may simply reflect a limitation of all ultrasound. Our findings support the conclusion by Montuori^9^ that current LUS techniques do not appear sensitive for cavity detection likely because of the challenges of interpreting the sonographic signs of air within a cavity, or because of many cavities not reaching the pleural surface. Further studies should evaluate alternate techniques that may improve cavity visualization.

The use of LUS has many potential advantages in low- and middle-income countries. It has a steep learning curve, is free of radiation exposure, and is available at a reasonable cost compared with other imaging modalities. Lung ultrasound is portable and can operate on rechargeable batteries without a continuous electricity supply. The only consumable supply needed is ultrasound gel, which can be locally produced.^24^ All these factors make it an attractive option in resource-limited settings.^25,26^ In addition to the potential utility of LUS for diagnosis of PTB, abdominal ultrasound is also a promising modality for diagnosis of abdominal or disseminated TB in HIV patients, especially in resource-limited settings.^26,27^ Ultrasound for TB diagnosis also has potential benefit in the pediatric population^28^ and could be of particular benefit in management of children with HIV in resource-limited settings.^29^

Future studies should compare LUS findings in PTB with those in other common pulmonary diseases, especially bacterial pneumonia, pneumocystis pneumonia, and cancer, to determine the ability of LUS to differentiate between these processes. In addition, radiographic sequelae of prior PTB infection, which are frequently localized in the apical regions, could represent a potential confounder, and this should be investigated in future studies.

Strengths of this study include a systematic LUS technique with data capture in 18 regions across the thorax, external expert review of LUS images with excellent agreement, and comparison of LUS findings to CXR interpreted by a board-certified radiologist. Study limitations include lack of a control group without PTB and the possibility for over-identification of LUS findings expected in PTB. Results from this proof-of-concept study suggest LUS has high potential as a sensitive method for diagnosis of PTB and could be particularly useful in settings where CXR is not possible.

In conclusion, an LUS composite of consolidation or SPC may be a sensitive marker for PTB. Lung ultrasound demonstrated poor ability to detect radiographically identified cavity, although previous studies suggest SPC might add specificity for the diagnosis of PTB. Based on its portability and evidence base for diagnosing other pulmonary disease, LUS may have a role in screening and diagnosis of PTB in areas without ready access to CXR. Further studies should evaluate its diagnostic accuracy in patients with and without PTB.

## References

[1] World Health Organization, 2019 Global Tuberculosis Report 2019. Geneva, Switzerland: WHO.

[2] Siddiqi K Lambert M-L Walley J, 2003 Clinical diagnosis of smear-negative pulmonary tuberculosis in low-income countries: the current evidence. Lancet Infect Dis 3: 288–296. 12726978 10.1016/s1473-3099(03)00609-1

[3] World Health Organization/Programme for Research and Training in Tropical Disease, 2013 Priorities for Tuberculosis Research: A Report of the Disease Reference Group Report on TB, Leprosy and Buruli Ulcer. Geneva, Switzerland: World Health Organization.

[4] Chavez MA 2014 Lung ultrasound for the diagnosis of pneumonia in adults: a systematic review and meta-analysis. Respir Res 15: 50. 24758612 10.1186/1465-9921-15-50 PMC4005846

[5] Llamas-Álvarez AM Tenza-Lozano EM Latour-Pérez J, 2017 Accuracy of lung ultrasonography in the diagnosis of pneumonia in adults. Chest 151: 374–382. 27818332 10.1016/j.chest.2016.10.039

[6] Ye X Xiao H Chen B Zhang S, 2015 Accuracy of lung ultrasonography versus chest radiography for the diagnosis of adult community-acquired pneumonia: review of the literature and meta-analysis. PLoS One 10: e0130066. 26107512 10.1371/journal.pone.0130066 PMC4479467

[7] Amatya Y Rupp J Russell FM Saunders J Bales B House DR, 2018 Diagnostic use of lung ultrasound compared to chest radiograph for suspected pneumonia in a resource-limited setting. Int J Emerg Med 11: 8. 29527652 10.1186/s12245-018-0170-2 PMC5845910

[8] Di Gennaro F 2018 Potential diagnostic properties of chest ultrasound in thoracic tuberculosis - a systematic review. Int J Environ Res Public Health 15: 2235. 10.3390/ijerph15102235 PMC6210728 30322009

[9] Montuori M Casella F Casazza G Franzetti F Pini P Invernizzi C Torzillo D Rizzardini G Galli M Cogliati C, 2019 Lung ultrasonography in pulmonary tuberculosis: a pilot study on diagnostic accuracy in a high-risk population. Eur J Intern Med 66: 29–34. 31235198 10.1016/j.ejim.2019.06.002

[10] Agostinis P Copetti R Lapini L Monteiro GB Deque AN Baritussio A, 2017 Chest ultrasound findings in pulmonary tuberculosis. Trop Doct 47: 320–328. 28541140 10.1177/0049475517709633

[11] Heuvelings CC Bélard S Andronikou S Lederman H Moodley H Grobusch MP Zar HJ, 2019 Chest ultrasound compared to chest X‐ray for pediatric pulmonary tuberculosis. Pediatr Pulmonol 54: 1914–1920. 31475477 10.1002/ppul.24500 PMC6899616

[12] Cozzi D Gianelli F Moroni C, 2019 Lung Ultrasound in Pulmonary Tuberculosis: Preliminary Results. Poster Abstract presented at ESTI-Fleischner 2019 Joint Meeting, May 2019, Paris, France. 10.26044/esti2019/P-0062.

[13] World Health Organization, 2017 Peru Country Profile, Tuberculosis. Geneva, Switzerland: WHO.

[14] Reissig A Copetti R Mathis G Mempel C Schuler A Zechner P Aliberti S Neumann R Kroegel C Hoyer H, 2012 Lung ultrasound in the diagnosis and follow-up of community-acquired pneumonia: a prospective, multicenter, diagnostic accuracy study. Chest 142: 965–972. 22700780 10.1378/chest.12-0364

[15] Requena-Méndez A Aldasoro E Muñoz J Moore DAJ, 2015 Robust and reproducible quantification of the extent of chest radiographic abnormalities (and it’s free!). PLoS One 10: 1–7. 10.1371/journal.pone.0128044 PMC4440724 25996917

[16] van Cleeff MRA Kivihya-Ndugga LE Meme H Odhiambo JA Klatser PR, 2005 The role and performance of chest X-ray for the diagnosis of tuberculosis: a cost-effectiveness analysis in Nairobi, Kenya. BMC Infect Dis 5: 111. 16343340 10.1186/1471-2334-5-111 PMC1326228

[17] Den Boon S White NW Van Lill SWP Borgdorff MW Verver S Lombard CJ Bateman ED Irusen E Enarson DA Beyers N, 2006 An evaluation of symptom and chest radiographic screening in tuberculosis prevalence surveys. Int J Tuberc Lung Dis 10: 876–882. 16898372

[18] Gargani L Volpicelli G, 2014 How I do it: lung ultrasound. Cardiovasc Ultrasound 12: 25. 24993976 10.1186/1476-7120-12-25 PMC4098927

[19] Lichtenstein DA Mezière GA, 2008 Relevance of lung ultrasound in the diagnosis of acute respiratory failure * the BLUE protocol. Chest 134: 117–125. 18403664 10.1378/chest.07-2800 PMC3734893

[20] Lichtenstein DA Lascols N Mezière G Gepner A, 2004 Ultrasound diagnosis of alveolar consolidation in the critically ill. Intensive Care Med 30: 276–281. 14722643 10.1007/s00134-003-2075-6

[21] Hunter L Bélard S Janssen S van Hoving DJ Heller T, 2016 Miliary tuberculosis: sonographic pattern in chest ultrasound. Infection 44: 243–246. 26661658 10.1007/s15010-015-0865-8

[22] Heuvelings CC Bélard S Janssen S Wallrauch C Grobusch MP Brunetti E Giordani MT Heller T, 2016 Chest ultrasonography in patients with HIV: a case series and review of the literature. Infection 44: 1–10. 25972115 10.1007/s15010-015-0780-z PMC4735240

[23] Volpicelli G 2012 International evidence-based recommendations for point-of-care lung ultrasound. Intensive Care Med 38: 577–591. 22392031 10.1007/s00134-012-2513-4

[24] Salmon M Salmon C Bissinger A Muller MM Gebreyesus A Geremew H Wende SK Azaza A Salumu M Benfield N, 2015 Alternative ultrasound gel for a sustainable ultrasound program: application of human centered design. PLoS One 10: e0134332. 26252003 10.1371/journal.pone.0134332 PMC4529075

[25] Heller T Mtemang’ombe EA Huson MAM Heuvelings CC Bélard S Janssen S Phiri S Grobusch MP, 2017 Ultrasound for patients in a high HIV/tuberculosis prevalence setting: a needs assessment and review of focused applications for sub-Saharan Africa. Int J Infect Dis 56: 229–236. 27836795 10.1016/j.ijid.2016.11.001

[26] Stefan W Kavitha S Tom H Rajagopal K Shashidhar V Stephan G Bélard S, And Pocus Eti Study Group, 2017 Point-of-care ultrasound for extra-pulmonary tuberculosis in India: a prospective cohort study in HIV-positive and HIV-negative presumptive tuberculosis patients. Am J Trop Med Hyg 98: 266–273. 10.4269/ajtmh.17-0486 PMC5928723 29141727

[27] Bobbio F Di Gennaro F Marotta C Kok J Akec G Norbis L Saracino A Mazzucco W Lunardi M, 2019 Focused ultrasound to diagnose HIV-Associated tuberculosis (FASH) in the extremely resource-limited setting of South Sudan: a cross-sectional study. BMJ Open 9: 1–7. 10.1136/bmjopen-2018-027179 PMC6500283 30944140

[28] Bélard S Heuvelings CC Banderker E Bateman L Heller T Andronikou S Workman L Grobusch MP Zar HJ, 2017 Utility of point-of-care ultrasound in children with pulmonary tuberculosis. Pediatr Infect Dis J 37: 637–642. 10.1097/INF.0000000000001872 PMC5995614 29278611

[29] Marotta C 2018 Pathways of care for HIV infected children in Beira, Mozambique: pre-post intervention study to assess impact of task shifting. BMC Public Health 18: 1–9. 10.1186/s12889-018-5646-8 PMC5992883 29879951

